# Fat Folks

**Published:** 1876-07

**Authors:** 


### *FAT FOLKS.*

Fat, when excessive, is disease, degeneracy. To lessen it, the conditions and habits which induce it must be changed. The body, if indolent, as is usual in this disease, must become active. The food, if fat making, must be reformed. The use of malt liquors and sacharine wines must be avoided. In short, there must be a radical and yet an intelligent change in all the conditions which have induced the unfortunate state.

Yet starvation must not occur. The "Banting" system was favorable to a lessening of adipose, because it cut off many of the sources of supply. It accomplished some results, for the reason that it afforded the disease of fatness less fuel with which to feed its flames. But it was radically wrong, and injurious, in that it did not supply strong nutriment in place of the fat-producing substances withdrawn. Many who adopted it were cured, and many were killed. There were radical errors in the system, and hence it fell into disuse. Taking its results together, so far as we have observed them, it probably did quite as much harm as good.

The disease of fatness, obesity, corpulence, is a serious matter, vastly more serious than the multitude of fat men and women—who bear their fearful burden of deformity toilsomely from place to place—are willing to concede. They know that life is weakened in all its functions, that they are not strong, that small exertions distress them, that strange feelings of heart and lungs and brain oppress them from time to time. But they rarely reflect that these symptoms mean anything serious. Far less do they consider that they are in reality the victims of a very serious chronic disease, which, unless arrested, must greatly lessen bodily vigor and seriously shorten life.

There is a rotundity provided for as life advances, which is natural and salutary. It is the moderate storing up of adipose to provide warmth in declining years. It is the added garment supplied by thoughtful nature for the protection of the less ardent blood. It is the gracious padding with which the wasted muscles are rounded out to hide to the last the encroachments of age. This is a condition to be desired rather than avoided. In a perfect state of health, this moderate rotundity is almost certain to ensue. If it does not appear, it is probable that there is either innutrition or a concealed drain upon the system. The most common form of innutrition is a lack of the proper quantity or kind of nerve food. Starvation of the nerves is a very dangerous variety of starvation. The drain upon the vitality may be some local disease, or some bad habit, or some sort of over work, or over indulgence. With suitable nerve food supplied in the one case, and the bad habit or reducing disease cured in the other, the attenuated state may be set aside and the kindly intentions of nature may be fulfilled.

The rotund condition natural to advancing years is not a state to be avoided, but rather encouraged. It is the excessive fatness which all sufferers should endeavor to avoid and remedy. The cure, the diminution of bulk when once established, is the condition to be sought. In this work there should be no empiricism, no quackery. If the person suffering from the disease of fatness is advised to drink vinegar, or eat slate-pencils, or, chew or smoke tobacco, to reduce the fat, let him decline so to do. Let him maintain as perfect a digestion as possible, but let him beware of the use of fat-making foods. Let him avoid starch—whether as potatoes, or flour bread, or rice. Let him abjure spirits and malt liquors. Let him carefully eschew sweets and fats. One food he may eat to advantage—the gluten of wheat. This substance will impart strength, but it will make no fat. It will sustain life in full vigor, but it will not add an ounce to the weight. It will compel activity of digestion, and if constipation exists, it will vanish. With the abolition of fat-forming elements, the adipose deposite terminates. The fuel demanded by the body, the body itself is compelled to supply. The fat induced by disease is slowly exhausted, and normal size and normal weight result. After that, right living is ample to secure freedom from the dangerous disease.

No class of citizens need advice more than do the fat folks, for none are in greater danger of early death, or lingering misery. In behalf of these we are glad to see that the Health Food Company are exerting themselves with gratifying results. We are convinced that the victims of fatty degeneracy can generally be restored to health and naturalness by following the advice of this Company, whom we regard as the highest authority on food topics on this continent.

